# Long-term diagnostic stability, predictors of diagnostic change, and time until diagnostic change of first-episode psychosis: a 21-year follow-up study

**DOI:** 10.1192/j.eurpsy.2025.1815

**Published:** 2025-08-26

**Authors:** D. P. Donner, L. Janda, E. Garcia de Jalon, L. Moreno-Izco, A. M. Sanchez-Torres, M. J. Cuesta, V. Peralta

**Affiliations:** 1 Mental Health Department, Servicio Navarro de Salud; 2 Department of Psychiatry, Instituto de Investigacion Sanitaria de Navarra (IdiSNA); 3 Department of Psychiatry, Hospital Universitario de Navarra, Pamplona, Spain

## Abstract

**Introduction:**

Although diagnostic instability in first-episode psychosis (FEP) is of major concern, little is known about its determinants.

**Objectives:**

To examine the long-term diagnostic stability of FEP diagnoses, the baseline predictors of diagnostic change to schizophrenia and the timing of diagnostic change.

**Methods:**

This was a longitudinal and naturalistic study of 243 subjects with FEP who were assessed at baseline and reassessed after a mean follow-up of 21 years. The diagnostic stability of DSM-5 psychotic disorders was examined using prospective and retrospective consistencies, logistic regression was used to establish the predictors of diagnostic change to schizophrenia, and survival analysis was used to compare time to diagnostic change across diagnostic categories.

**Results:**

The overall diagnostic stability was 47.7%. Schizophrenia and bipolar disorder were the most stable diagnoses, with other categories having low stability. Predictors of diagnostic change to schizophrenia included a family history of schizophrenia, obstetric complications, developmental delay, childhood adversity, poor premorbid functioning in several domains, long duration of untreated continuous psychosis, spontaneous dyskinesia, lack of psychosocial stressors, and poor early treatment response (Table 1). There were no significant differences between specific diagnoses regarding time to diagnostic change. At 10-year follow-up, around 80% of the diagnoses had changed.

Table 1. Main baseline predictors of diagnostic change to schizophrenia over the follow-up (univariate logistic regression)

**Table:** 

	No diagnostic change (n=124) ^†^	Diagnostic change (n=47) ^†^	OR (95% CI)	p
Family history of schizophrenia spectrum disorders	16 (12.9)	16 (34.0)	3.48 (1.56 – 7.75)	**0.002**
Obstetric complications, any definite	9 (7.3)	12 (25.5)	4.38 (1.70 – 11.2)	**0.002**
Developmental delay at year 3, any	30 (24.2)	30 (63.8)	5.52 (2.68 – 11.3)	**<0.001**
Childhood adversity score, high (< 77)	39 (31.5)	24 (55.1)	2.27 (1.14 – 4.51)	**0.019**
Premorbid adjustment score, poor (≥ 4)	30 (24.2)	23 (48.9)	3.00 (1.48 – 7.07)	**0.002**
Acute psychosocial stressors, any	62 (50.0)	11 (23.4)	0.30 (0.14 – 0.65)	**0.002**
Duration of untreated continous psychosis, long (≥ 1 month)	34 (27.4)	26 (55.3)	3.27 (1.63 – 6.58)	**0.001**
Spontaneous dyskinesia, Schooler & Kane criteria	2 (1.9)	9 (24.3)	17.0 (3.48 – 83.3)	**<0.001**
CGI-EI at index discharge, marked improvement	100 (80.6)	28 (59.6)	0.35 (0.17 – 0.73)	**0.005**

† Data are number (and percentages) of the stated features

CGI-EI = Clinical global impression-Efficacy Index

**Conclusions:**

FEP diagnoses other than schizophrenia or bipolar disorder should be considered as provisional. Considering baseline and background predictors of diagnostic change to schizophrenia may help to enhance diagnostic accuracy and guide therapeutic interventions.

**Disclosure of Interest:**

None Declared

